# Direction to an Internet Support Group Compared With Online Expressive Writing for People With Depression And Anxiety: A Randomized Trial

**DOI:** 10.2196/mental.5133

**Published:** 2016-05-17

**Authors:** Jeremy Dean, Henry WW Potts, Chris Barker

**Affiliations:** ^1^ Department of Clinical, Educational, and Health Psychology University College London London United Kingdom; ^2^ Institute of Health Informatics University College London London United Kingdom

**Keywords:** depression, anxiety, Internet support, online support, expressive writing

## Abstract

**Background:**

Depression and anxiety are common, often comorbid, conditions, and Internet support groups for them are well used. However, little rigorous research has been conducted on the outcome of these groups.

**Objective:**

This study aimed to evaluate the efficacy of an Internet support group in reducing depression and anxiety, and increasing social support and life satisfaction.

**Methods:**

A randomized trial compared direction to an existing Internet support group for depression and anxiety with an online expressive writing condition. A total of 863 (628 female) United Kingdom, United States, and Canadian volunteers were recruited via the Internet. Online, self-report measures of depression, anxiety, social support, and satisfaction with life were administered at baseline, 3, and 6 months.

**Results:**

All four outcomes – depression, anxiety, social support, and satisfaction with life – improved over the 6 months of the study (all *P*<.001). There was no difference in outcome between the two conditions: participants responded similarly to the expressive writing and the Internet support group. Engagement with the Internet support group was low, it had high 6-month attrition (692/795, 87%) and low adherence, and it received mixed and often negative feedback. The main problems reported were a lack of comfort and connection with others, negative social comparisons, and the potential for receiving bad advice. Expressive writing had lower attrition (194/295, 65%) and participants reported that it was more acceptable.

**Conclusions:**

Until further evidence accumulates, directing people with depression and anxiety to Internet support groups cannot be recommended. On the other hand, online expressive writing seems to have potential, and its use for people with depression and anxiety warrants further investigation.

**Trial Registration:**

Trial Registration: Clinicaltrials.gov NCT01149265; https://clinicaltrials.gov/ct2/show/NCT01149265 (Archived by WebCite at http://www.webcitation.org/6hYISlNFT)

## Introduction

Internet support groups (ISGs) covering health and well-being are undoubtedly popular: tens of millions of people have joined them in the United States alone [1,2]. Barak et al. [3] estimate that there may be several hundred thousand of these groups. Many focus on mental health problems, particularly depression and anxiety, which are prevalent and persistent disorders. A review of European studies estimated that the 1-year prevalence of major depressive disorder was 5.7% [4], and it is frequently comorbid with anxiety [5]. However, for various reasons, people with depression and anxiety may be reluctant to seek formal psychological help [6], and so ISGs represent a potential additional source of informal help for them [7].

Given the prevalence of depression and anxiety and the popularity of online support, it is important that studies be carried out to estimate the overall outcome of ISGs, and to understand the mechanisms that may account for any beneficial effects. Evidence from these studies will help people with depression and anxiety decide whether an ISG is worth joining, and help professionals decide whether ISGs might benefit their patients. ISGs could potentially be an adjunct to other types of more intensive psychological treatment like cognitive-behavioral therapy or be a standalone low-intensity intervention in their own right.

There has been relatively little work on the outcome of ISGs for common mental health problems. Griffiths et al [7] reviewed studies of ISGs that measured depression as an outcome (not necessarily in ISGs primarily for people with depression). The majority of the 17 studies reviewed found positive effects on depression, although only two [8,9] were of depression-specific ISGs. Griffiths et al’s randomized controlled trial (RCT) [10] compared a purpose-built ISG for depression with a computerized cognitive behavioral therapy condition, and found that the ISG participants had generally better outcomes at 6 and 12 months (but not 3 months).

The present study investigated the effectiveness of directing individuals to an existing ISG for depression and anxiety. It was partly motivated by the National Institute for Clinical Excellence (NICE) guidelines [11], which recommend the development of accessible help and support for people with common mental health problems. The design was a 6-month RCT. Participants in the experimental condition were facilitated to join the forums on a specific ISG, chosen because it had a constructive atmosphere and high traffic. Such an intervention mimics the approach that health care professionals or online resources can take, of directing those interested to existing groups, rather than the approach taken in some studies [10,12] where a group is created for the purposes of the study.

The selection of a comparison group for a naturalistic ISG study is problematic. Traditional waitlist control groups are difficult to maintain, as participants, prompted by their involvement in a study, can simply find and use other Web-based resources. We therefore chose an active control group, consisting of an online expressive writing intervention [13,14], which involves asking participants to write about "a traumatic experience" for between 15 and 20 minutes per day over a period of 3 to 5 days. A meta-analysis [15] found that expressive writing was effective in reducing psychological distress and increasing physical health, although the aggregated effect size was very small: 0.075. There are also similarities between expressive writing and the interactions between ISG users: both involve the expression of upsetting thoughts and emotions, although in expressive writing the writing is addressed to the self, whereas in an ISG it is addressed to the online community. However, there are additional therapeutic benefits potentially present in an ISG but not in expressive writing, such as receiving both information and emotional support from fellow group members, plus the sense of normalization of one’s difficulties and the instillation of hope [16].

The main hypothesis was that participants randomized to the ISG condition would accordingly show greater improvement on the primary outcome measures (depression and anxiety) than those in the expressive writing condition. Secondary outcomes were perceived social support and satisfaction with life. Because increased social support is one of the mechanisms by which ISGs are thought to benefit their users, in accordance with Houston et al [9] it was hypothesized that social support would improve over time. Following Freeman et al [12], we hypothesized that a successful intervention would not only decrease depression and anxiety, but also increase satisfaction with life. These latter two outcome variables, social support and life satisfaction, have also been found to be correlated among users of social network sites [17].

In addition, a word count analysis was conducted to examine the associations between the language used in ISG postings and changes in depression over time. Following Pennebaker and Francis [18] and Riessman [19] respectively, we hypothesized that participants who (1) expressed more positive and negative emotions, and (2) used more other-focused pronouns would tend to have a greater reduction in depression.

## Methods

The study was a CONSORT-R compliant RCT [20]. The protocol was registered with clinicaltrials.gov, a database of clinical trials run by the US National Institute of Health [trial ID: NCT01149265].

### Design

The design was a 6-month RCT with participants randomized to either (1) direction to an ISG, or (2) an expressive writing condition. Measurement points were baseline, 3, and 6 months. Participants were randomized in a 2:1 ratio in favor of the ISG condition (because a pilot study found that attrition was twice as great in that condition).

### Recruitment

To recruit a Web-based sample, adverts were placed on a popular website, PsyBlog [21], run by the first author. Other individuals and organizations also publicized the study through websites, Facebook, and Twitter. Table 1 shows how participants located the study. Recruitment occurred between April and July 2010.

**Table 1:** How participants located the study.

Source	N (%)
Twitter	412 (48%)
PsyBlog	187 (22%)
Google search	58 (7%)
Facebook	56 (6%)
Discussion forum	36 (4%)
Other	116 (13%)
Total	863 (100%)

The inclusion criteria were that participants were (1) over 18, (2) able to access the Internet, (3) English-speaking and living in the United Kingdom, the United States, or Canada, (4) experiencing self-defined depression or anxiety, and (5) computer literate. There were 1192 participants who met the inclusion criteria (see the Results section for their characteristics). Applicants who did not meet the criteria were sent an email thanking them for their interest.

The study was approved by the University Research Ethics Committee. Web-based informed consent was obtained from all participants.

### Interventions

#### Internet Support Group Condition

Participants were randomized to either direction to an ISG or to the expressive writing condition. Participants in the ISG condition were asked to take part in the existing groups hosted at Psych Central [22], and given instructions on how to register and choose a username, password, and screen name. They were instructed to not use a screen name that personally identified them. They were shown the frequently asked questions page at the Psych Central forums and asked to familiarize themselves with the terms and conditions, and were provided with a list of hints and tips produced by the researcher, which outlined the potential benefits and issues that they may face in the ISG. They were told that they could contact the researcher at any stage if they were having any problems. Participants were encouraged to post an introductory message in the ISG and to try to take part in the ongoing discussions or start their own threads. Participants entered the group in batches over several weeks.

#### Expressive Writing Condition

The expressive writing paradigm, developed by Pennebaker and Beall [14], involves participants writing about their thoughts and feelings, often upsetting ones, for a short period of time. In the current study, participants were asked to write about an upsetting experience for a minimum of 5 minutes, every 2 weeks, over the 6 months of the study. They were asked to carry out this task any time during the 2-week period and submit it securely through a study website.

#### Email Reminders

In both conditions, participants were each sent a reminder email every 2 weeks. In the ISG condition it reminded them to take part in the ISG as well as asking how much they had used it in the last 2 weeks. In the expressive writing condition, it reminded them to carry out the expressive writing task and contained instructions on how to submit it online.

#### Sample Size and Randomization

A power calculation suggested that 51 participants per group would provide sufficient power to detect a medium between-groups effect size (Cohen’s d=0.5). It was carried out on the Center for Epidemiologic Studies Depression Scale (CES-D) using the G*Power 3 computer program [23], specifying alpha at 5% and desired power at 80%. To reach a minimum of 51 participants per group, however, a much larger number of participants had to be recruited. Pilot work yielded expected attrition rates of approximately 90% in the ISG group and approximately 70% in the expressive writing group. Therefore, 1200 participants were recruited. Because of the greater attrition in the ISG group in the pilot study, randomization was carried out at a 2:1 ratio in favor of the ISG condition. It was carried out remotely by the second author, a statistician, using random numbers generated in Excel.

**Figure 1:**
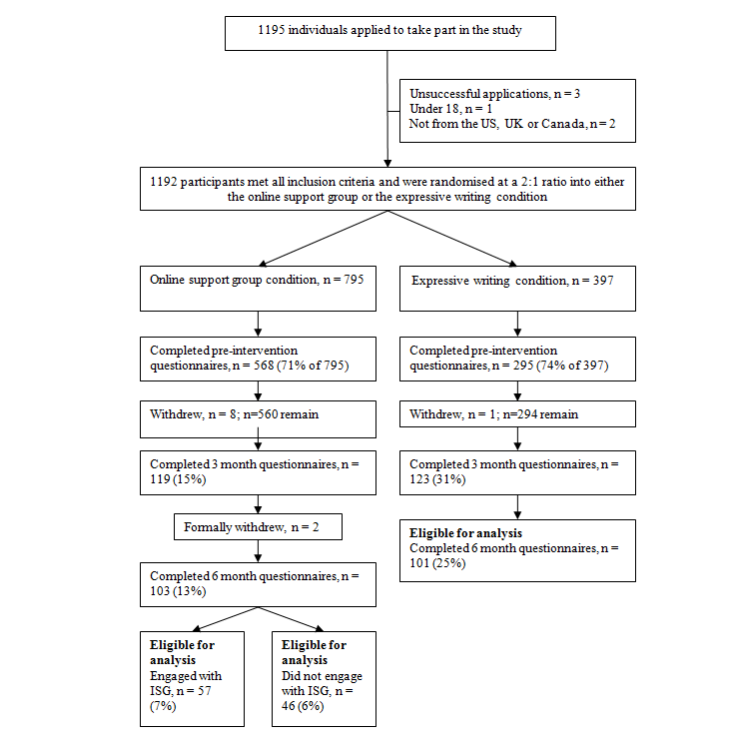
CONSORT-R participant flow chart.

Of the 863 participants in both conditions who completed the initial measures, 24% (204/863) completed the final measures after 6 months (see Figure 1).

### Outcome Measures

All measures were administered online using the Opinio software [24]. The primary outcome measure was the 20-item CES-D [25]. Items (eg, "[in the last week] I was bothered by things that usually don’t bother me") are rated on a 5-point scale from 1 = "Rarely or none of the time (less than 1 day)" to 5 = "Most or all of the time (5-7 days)". It has been validated for online use [26]. Cut-offs for depression vary between scores of 16 and 27 [25,27,28].

The General Anxiety Disorder Questionnaire (GAD-7) [29] is a 7-item anxiety scale. It asks how often in the last 2 weeks the respondent has felt worried or tense (eg, "Worrying too much about different things"). It is rated on a 4-point scale from 0 = "Not at all" to 3 = "Nearly every day". It has good psychometric properties (Cronbach's α = 0.92) [30].

The Medical Outcomes Study Social Support Survey (MOSSSS) [31] is a 19-item scale that assesses perceived functional social support. The items ask how often someone is available to give certain types of support (eg, "Someone to give you good advice about a crisis"), rated on a 5-point scale from 1 = "None of the time" to 5 = "All of the time". It has five subscales: emotional support, informational support, affection, tangible support, and positive interaction, with high internal consistency [31].

The Satisfaction with Life Scale (SWLS) [32] measures global satisfaction with life. It has five items (eg, “In most ways my life is close to ideal”) rated on a 7-point scale, from 1 = “Strongly disagree” to 7 = “Strongly agree.” It has good reliability and validity [33,34].

A slightly modified version of the Brief Illness Perception Questionnaire (IPQ) [35] was used to assess participants' expectations. The standard scale has nine items, five of which assess cognitive illness representations, two assess emotional representations, one assesses illness comprehensibility, and one assesses causal representations. Only five of these were used in the current study, in a slightly modified form to make them relevant for the study's participants. The items (eg, "How much does your condition [eg, depression, anxiety] affect your life?") were rated on an 11-point scale, from 0 = “No effect at all” to 10 = “Severely affects my life." The IPQ has good reliability and validity [36].

Participants' level of satisfaction with the ISG was measured at the end of the study using the Online Support Group Questionnaire [37], a nine-item scale, which measures satisfaction across three areas: comfort-connection, relevance, and support. Items (eg, "I felt satisfied with being part of the group") are rated on a seven-point scale ranging from 0 = “Not at all” to 7 = “Very much.” Good internal consistency and reliability has been reported for this measure [37].

### ISG Process Measures

#### Engagement

Participants' engagement with the ISG was assessed by asking them to report their usage every 2 weeks. First, they were asked how often they had accessed the ISG in the last 2 weeks. Responses were categorical, ranging from 0 = “Not in the last two weeks” to 5 = “More than 5 times.” Second, they were asked how long they had spent accessing the ISG on each occasion on a scale ranging from 0 = “Not applicable/never” to 5 = “More than 20 minutes.” Third, they were asked to report the number of messages they had posted in the last 2 weeks, on a scale ranging from 0 = “None” to 4 = “More than 5 times.”

#### ISG Posts

The text that participants wrote was collected from the ISG, with their permission, by using their anonymous usernames to search the ISG's forums. Although 57 participants were classified as engagers with the ISG based on self-reported use, it was only possible to collect data from 48, because nine participants’ usernames could not be matched with usage. For the 48 users for whom posts were available, a total of 1659 messages were posted across the 6 months of the study. However, a large number of posts were written by three participants, one of whom posted over 250 times. With these outliers included, the mean number of posts was 34.6. To avoid these three participants being too strongly represented, for those participants who had posted more than 32 times, their messages were randomly sampled to make 32 the maximum number of posts analyzed. This method led to a mean number of posts analyzed of 15 for each participant. In addition, some posts were excluded from the analysis: (1) posts to one of the forums on the ISG called 'Games', which consisted of word games, and (2) short replies to simple questions, such as "What is your favorite song?"

The text was cleaned up in Microsoft Word for analysis in the word counting software, Linguistic Inquiry and Word Count, version 1.08 (LIWC) [38]. The software uses a dictionary containing 86% of the words commonly used in speech and writing, placed into one or more of 64 categories, only a handful of which are relevant to the present study. These were positive and negative emotion words and the pronouns denoting either the first person (singular or plural) versus those denoting the second and third person (singular or plural). LIWC outputs the total number of words (as a percentage) that match the categories.

### Qualitative Data

After taking part in the study, as part of the final measures, which were collected online, participants were asked: "Finally, this last question is optional. If you like you can let us know what you thought of the online support group (expressive writing) and the study in general. You might like to tell us about both good and bad points. You might also like to suggest changes or improvements." There was a single free-text box for responses. Data were analyzed thematically [39]; coding was carried out using the Web-based software package Dedoose [40].

## Results

### Participants

At baseline, 863 participants (628/863 female; 73%) completed measures; of these 204 (157/204 female; 76%) completed the final measures at 6 months. Characteristics are given in Multimedia Appendix 1. The overall 76% (659/863) attrition rate was high, but it is comparable with similar Web-based studies [41]. Most participants did not indicate why they left the study. The attrition rate in the ISG condition was 85% (676/795) at 3 months and 87% (692/795) at 6 months; in the expressive writing condition it was 58% (172/295) at 3 months and 65% (194/295) at 6 months. The CONSORT-R flowchart [20] is given in Figure 1.

### ISG Engagement

ISG usage decreased markedly over time. The average frequency at which the ISG was accessed declined from twice every 2 weeks at the start down to less than once every 2 weeks by the end of the study. The average amount of time spent accessing the ISG declined from approximately 5 minutes in the first week to less than 1 minute toward the end. The number of posts participants made declined from approximately two in the first 2 weeks, down to almost zero by the week 12.

To analyze the characteristics of those who engaged with the ISG, an engager was defined as a participant who used the ISG on more than two occasions over the 6-month period. There were no differences on demographic variables between engagers and nonengagers. In particular, participants from the United States were no more likely to engage than those from the United Kingdom or Canada (χ^2^(2) = 0.70, *P*=0.71, N=103). Similarly no differences for engagement were seen for gender, age, ethnicity, education, whether participants were seeing a therapist or taking medication, and whether they had previously taken part in an online or face-to-face support group.

In terms of baseline outcome measures, engagers were more anxious (M=11.1, SD = 4.9) than nonengagers (M=7.8, SD = 5.6; t(101) = 3.2, *P*=0.002). For depression there was a similar trend with engagers’ CES-D scores marginally higher (M=30.3, SD = 11.8) than nonengagers (M=25.7, SD = 13.1; t(101) = 1.9, *P*=0.064). There were no differences for social support or satisfaction with life.

### Outcome: ISG Versus Expressive Writing

**Figure 2:**
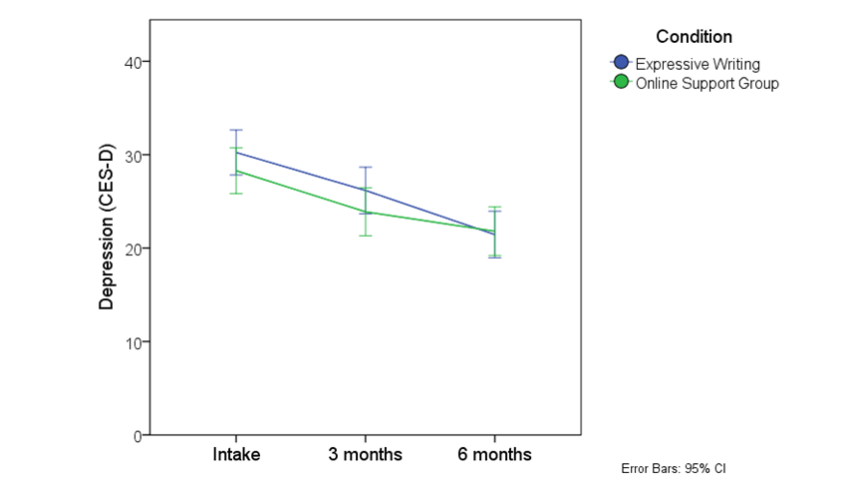
Mean CES-D scores at baseline, 3, and 6 months for all participants.

Following the study protocol, the initial analysis included all participants in the ISG and expressive writing conditions who completed the outcome measures at 6 months. Means and SDs for the four outcome measures are shown in Table 2. To assess the effects of using the ISG compared with carrying out the expressive writing task, a series of 3 (time, within groups) × 2 (condition, between groups) mixed analysis of variance (ANOVAs) were conducted. All four outcome variables showed a significant effect of time (depression: *F*_2,201_= 35.00, *P*<.001; social support: *F*_2,201_= 12.29, *P*<.001; satisfaction with life: *F*_2,201_= 16.67, *P*<.001; anxiety: *F*_2,201_= 13.39, *P*<.001) but none of the interaction effects was significant, suggesting there were no differences in the treatment effects between conditions (depression: *F*_2,201_= 1.57, *P*=0.21; social support: *F*_2,201_= 0.59, *P*=.56; satisfaction with life: F_2,201_= 0.19, *P*=.91; anxiety: *F*_2,201_= 1.09, *P*=.34). The marginal means for the CES-D are depicted in Figure 2 ; the other outcome variables showed a similar pattern.

**Table 2:** Outcome measures by condition.

Measure		Baseline M (SD)	3 months M (SD)	6 months M (SD)	Baseline-3 months effect size (95 CI)		Baseline-6 months, effect size (95 CI)
Depression (CES-D)							
	Expressive writing (n=101)	30.2 (12.2)	26.2 (12.7)	21.5 (12.7)	0.3 (0-0.6)		0.7 (0.4-1.0)
	ISG (n=103)	28.3 (12.5)	23.9 (13.2)	21.8 (13.3)	0.3 (0.1-0.6)		0.5 (0.2-0.8)
	Engagers (n=57)	30.3 (11.8)	26.1 (13.2)	23.6 (13.7)	0.3 (0-0.7)		0.5 (0.2-0.9)
	Nonengagers (n=46)	25.7 (13.1)	21.2 (12.7)	19.6 (12.7)	0.4 (0.1-0.8)		0.5 (0.1-0.9)
Social support (MOSSS)							
	Expressive writing (n=101)	50.9 (16.5)	52.1 (18.2)	54.3 (19.0)	-0.1 (-0.3-0.2)		-0.2 (-0.5-0.1)
	ISG (n=103)	55.1 (17.6)	57.4 (18.6)	60.4 (18.0)	-0.1 (0.4-0.2)		-0.3 (-0.6-0)
	Engagers (n=57)	52.4 (17.3)	54.8 (18.4)	59.5 (18.4)	-0.1 (-0.5-0.2)		-0.4 (-0.8-0)
	Nonengagers (n=46)	58.4 (17.6)	60.6 (18.6)	61.4 (17.6)	-0.1 (-0.5-0.3)		-0.2 (-0.6-0.2)
Satisfaction with life (SWLS)							
	Expressive writing (n=101)	14.7 (6.9)	15.7 (7.7)	17.0 (7.0)	-0.1 (-0.4-0.1)		-0.3 (-0.6 -0.1)
	ISG (n=103)	15.8 (7.5)	16.9 (8.2)	17.8 (8.0)	-0.1 (-0.4-0.1)		-0.3 (-0.5-0)
	Engagers (n=57)	15.5 (8.0)	16.9 (8.8)	17.8 (8.4)	-0.2 (-0.5-0.2)		-0.3 (-0.7-0.1)
	Nonengagers (n=46)	16.2 (7.0)	16.8 (7.4)	17.9 (7.5)	-0.1 (-0.5-0.3)		-0.2 (-0.6-0.2)
Anxiety (GAD-7)							
	Expressive writing (n=101)	9.8 (5.0)	9.0 (5.4)	7.6 (5.0)		0.2 (-0.1-0.4)	0.4 (0.2-0.7)
	ISG (n=103)	9.6 (5.5)	8.4 (5.5)	7.9 (5.8)		0.2 (0-0.5)	0.3 (0-0.6)
	Engagers (n=57)	11.1 (4.9)	9.4 (5.1)	8.6 (5.5)		0.3 (0-0.7)	0.5 (0.1-0.9)
	Nonengagers (n=46)	7.8 (5.6)	7.1 (5.7)	7.2 (6.1)		0.1 (-0.3-0.5)	0.1 (-0.3-0.5)

### Outcome: Engagers Versus Expressive Writing

**Figure 3:**
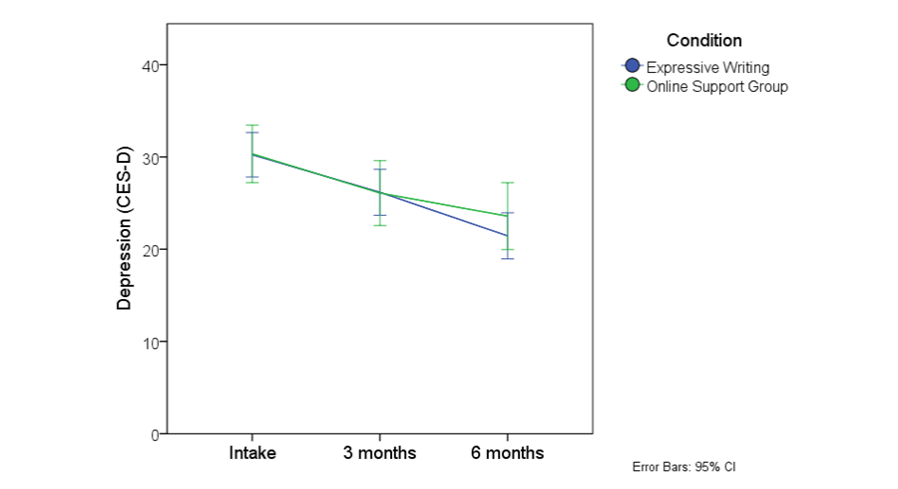
Mean depression scores on the CES-D at baseline, three, and six months excluding those who did not engage with the ISG.

A secondary analysis excluded nonengagers, comparing the 57 engagers in the ISG with all 101 expressive writing participants. A series of 3 (time, within groups) × 2 (condition, between groups) mixed ANOVAs were conducted. The pattern of results was the same as with the previous analysis. All four outcome variables showed a significant effect of time (depression: *F*_2,155_= 26.80, *P*<.001; social support: *F*_2,155_= 14.70, *P*<.001; satisfaction with life: F_2,155_= 14.05, *P*<.001; anxiety: *F*_2,155_= 15.74, *P*<.001) but none of the interaction effects were significant, suggesting there were no differences in the treatment effects between conditions (depression: *F*_2,155_= 0.78, *P*=.46; social support: *F*_2,155_= 1.88, *P*=.16; satisfaction with life: *F*_2,155_= 0.12, *P*=.88; anxiety: *F*_2,155_= 0.77, *P*=.46). The marginal means for the CES-D are depicted in Figure 3 ; the other outcome variables showed a similar pattern.

### Intention-to-Treat Analysis

Finally, an intention-to-treat analysis was also carried out, for all participants with baseline scores, using the last observation carried forward procedure. The results are not reported in detail as they showed a similar pattern to the previous analyses. There was no evidence that participants in the ISG condition experienced improved outcomes over the expressive writing condition.

### Expectations Analysis

Changes in expectation of the intervention’s usefulness over time, were analyzed in a series of 3 (time, within groups) × 2 (condition, between groups) mixed ANOVAs. There were effects of time on expectations of "the condition's influence on life" (*F*_2,201_= 6.1, *P*=.003), "control over the condition" (*F*_2,201_= 8.24, *P*<.001) and "expectations of the intervention's use" (*F*_2,201_= 13.21, *P*<.001), but not on "expected longevity of the condition" (*F*_2,201_= 1.98, *P*=.14), or "understanding of condition" (*F*_2,201_= 1.49, *P*=.23). Two of the effects were in a psychologically positive direction (ie, toward more control and lower effect of the condition on life but expectations of the intervention's usefulness declined). The interaction was only significant for expectations of the intervention's use (*F*_2,201_= 16.69, *P*<.001), suggesting expectations changed differentially in each group, so this was further explored.

**Figure 4:**
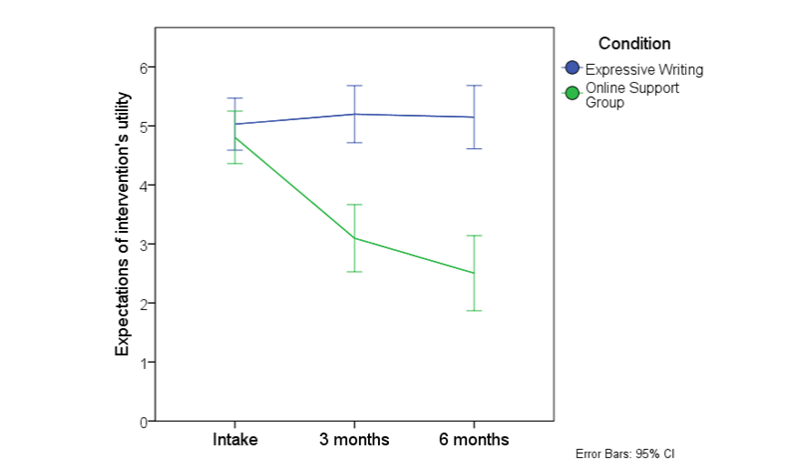
Expectation of the intervention's utility at baseline, three and six months for all participants eligible for analysis.

A plot of the means (Figure 4) for expectations of the intervention's usefulness suggested that the source of the interaction was a drop in expectations over time in the ISG condition and not the expressive writing condition. A one-way ANOVA conducted on the expectation scores on the ISG group suggested that expectations had changed over time (*F_2,608_* = 8.69, *P*<.001). Post-hoc tests using the least significant difference (LSD) correction for multiple comparisons revealed a drop in expectations between baseline (M=4.9, SD = 2.2) and 3 months (M=4.1, SD = 2.9; *P*=.04) and between baseline and 6 months (M=3.8, SD = 3.2; *P*<.001).

### Satisfaction Data

Table 3 shows the means for each of the nine items of the OSGQ [37]. The satisfaction levels in each of the categories for engagers are clustered around the midpoint of the scale, except for anonymity, which is higher. The satisfaction of the nonengagers was significantly lower on every variable, but, again, the importance of anonymity is underlined.

### Word Count Analysis

Forty-eight participants who engaged with the ISG and who provided matchable username information were included in the linguistic analysis. In total, they posted 1659 messages across the full 6 months of the study. As described above, messages from three prolific posters were randomly sampled, leaving a total of 722. Messages ranged in length from 38 to 6124 words, with the total analyzed being 91,084.

To analyze the associations between ISG language use and improvement, correlations were carried out between the improvements on the outcome measures and the features of language use. Improvement was calculated by the difference between baseline scores and those at 3 and 6 months. The categories of language use tested were positive and negative emotions and the use of first-person singular pronouns and second- and third-person pronouns. The aim was to test the degree to which participants were talking about themselves, compared with interacting with others. Spearman correlations were carried out as the word count data were not normally distributed. For the 3-month data, only one of the correlations was significant: that between improvement on depression scores and the expression of positive emotions (Table 4). For the 6-month data, only one correlation was significant, that between improvement in social support and use of the "I" pronoun (Table 5). There was, therefore, only weak evidence for the first directional hypothesis and no support for the second hypothesis that improvements in depression would be associated with higher use of second-person pronouns.

**Table 3:** Satisfaction with the ISG.

	Engagers (n=57)	Non-engagers (n=46)	t(101)	*P*
Variable	M (SD)	M (SD)		
Felt supported	3.60 (2.62)	0.78 (1.85)	6.16	<.001
Felt listened to	3.40 (2.52)	0.67 (1.96)	6.02	<.001
Relevance of discussion	3.81 (2.19)	0.54 (1.39)	8.79	<.001
Others addressed my issues	3.39 (2.39)	0.39 (1.37)	7.56	<.001
Comfortable raising issues	3.33 (2.63)	0.78 (1.76)	5.63	<.001
Connection to other members	2.44 (1.84)	0.61 (1.47)	5.48	<.001
Satisfied with group membership	2.95 (2.26)	0.70 (1.72)	5.57	<.001
Importance of anonymity	5.12 (2.56)	2.20 (3.14)	5.20	<.001

**Table 4:** Spearman correlations between improvement on outcome measures and facets of language use in the ISG over the first 3 months.

Outcome	Positive emotion	Negative emotion	"I"	"We, you, he, she, and they"
Depression	.38^a^	.02	.25	.09
Social support	.27	-.25	-.13	.11
Satisfaction with life	.21	-.07	-.04	.15
Anxiety	.27	-.11	-.17	.13

^*^ *P*=.009

**Table 5:** Spearman correlations between improvement on outcome measures and facets of language use in the ISG over the full 6 months of the study.

Outcome	Positive emotion	Negative emotion	"I"	"We, you, he, she, and they"
Depression	.01	-.13	.25	.09
Social support	-.09	.04	.31^*^	.11
Satisfaction with life	.06	-.15	.08	-.03
Anxiety	0	-.21	-.02	.15

^*^ *P*=.03

### Qualitative Data

In the ISG condition, 73 participants wrote free-text comments after the intervention

The analysis yielded four main themes: (1) comfort and connection, (2) social comparisons, (3) needing guidance, and (4) advice. Overall, the responses were quite negative for most of the themes, with people pointing out more problems than beneficial aspects (see Textbox 1 for subthemes and illustrative quotations).

In the expressive writing condition, 69 participants provided comments. The analysis yielded two main themes: (1) emotional effects and (2) task tweaks. The feedback was predominantly positive, with many participants pointing out the beneficial aspects of the writing task (see Textbox 2).

In Textboxes 1 and 2 a typical theme is one which applies to more than one-half of the participants, a variant theme applies to up to one-half of participants, and a rare theme applies to less than one-tenth of participants.

**Themes and subthemes in participants' ISG feedback**

Themes and subthemes (frequency) with an illustrative quote:

Comfort and connection

- Negative: the ISG is too big (variant): “ *I found it overwhelming trying to settle into a place to go, and then how to respond. There was an overload of people, problems and information.”* (P48)
- Negative: unsupportive (variant): “ *I think not getting involved was healthier for me as, frankly, most of the threads I read were people winding each other up and making each other more anxious.”* (P55)
- Positive: warm and supportive (variant): “ *The forum used for this study was very friendly and usually answered my posts and seemed appreciative of my responses.”* (P8)

Social comparisons

- Negative: they are not like me (variant): *I was afraid to talk about my problems because it felt like nobody else had ever dealt with the same thing. It almost felt like talking about it in the group was worse than dealing with it on my own.”* (P45)
- Negative: triggers (variant): “ *Reading posts by other people often triggered a negative feeling for me, and made me feel more anxious about myself.”* (P11)
- Negative: my trivial problems (variant): “ *It seemed that the majority of the regular posters on Psych Central went way beyond a tad anxious or a bit blue. A lot of the members had severe mental illnesses or told stories about going through horrendously traumatic experiences. I felt a little over my head in the community.”* (P22)
- Positive: putting it into perspective (rare): “ *I do appreciate that this group exists for people with a much more severe "condition" than mine and it is good to know it is here.”* (P51)

Need guidance using the site (rare)

- *“I had no idea how to start as I was depressed.”* (P2)

Advice

- Negative: bad advice (variant): “ *There seemed to be a hell of a lot of ill-informed rubbish posted, which could - in the case of medication or treatment - be dangerous.”* (P55)
- Positive: good advice (rare): “ *There were some very interesting discussions raised over the last few months, which have helped me look at my illness and recovery in a different way.”* (P62)

**Themes and subthemes in participants' expressive writing feedback**

Themes and subthemes (frequency) with an illustrative quote:

Emotional effects

- Positive: feeling better (typical): “ *The activity itself was very uplifting. I felt I had gotten a huge weight off my shoulders. I feel that this was a very effective way of alleviating what I feel was a moderate (but still significant) level of depression and anxiety due to a combination of genetics, environment, and the usual lark.”* (P55)
- Negative: feeling worse (rare): “ *Sometimes doing the writing and the questionnaires made me feel more depressed and anxious than if I wasn't thinking about those things.”* (P60)

Task tweaks

- Lack of feedback (rare): “ *I felt I was still totally on my own, there was no response, there was no indication that anyone was even interested in my thoughts let alone reading them.”* (P34)
- Writing prompts (rare): “ *I would have preferred boxes with headings to fill in I think-a blank box to ramble on in to be read by unknown people didn't feel very constructive.”* (P36)
- Positive writing (rare): “ *While expressive writing was helpful, and I do think it's important to explore the bad, it might be more helpful to also explore the good. To remind myself that it's not always bad.”* (P21)

## Discussion

### Principal Findings

The study aimed to test the effectiveness of an ISG for depression and anxiety by comparing it with an expressive writing intervention, thereby extending previous research, which has not involved a comparison group. When all participants eligible for analysis were included, all four outcomes – depression, anxiety, social support, and satisfaction with life – improved over the 6 months of the study. However, there were no differences in outcome between the ISG and the expressive writing conditions, although the expressive writing group had lower attrition, better engagement, and more positive user feedback. We must therefore conclude that the ISG intervention provided no additional benefit to that obtained by expressive writing. That may be because the ISG intervention was weaker than predicted, or because the expressive writing condition was stronger than predicted. We explore both possibilities below.

The low rate of engagement and high rate of attrition suggest that the ISG intervention was not a strong or attractive one. Either our direction to participants was insufficient to motivate their engagement with the ISG, or the ISG did not suit our participants, a possibility which the qualitative data seem to support. It is likely that its impact was diluted by this lack of engagement. However, an analysis only including engagers still showed no differences between the conditions. We set our definition of engagers quite low, but the number of high engagers in the study was small. It is possible that the ISG selected for the study was not typical or in some way less effective, at least for our participants. However, we put considerable effort into choosing an existing ISG for the study, and Psych Central appeared then, and appears now, to be one of the best available.

The second possibility is that the expressive writing task had a stronger than expected impact. We picked expressive writing to be a plausible comparator, but we did not expect it to be of as much benefit as the ISG. However, it was certainly more attractive to participants, with notably lower attrition and more positive user feedback. The effect size found for expressive writing over time in this study is above the average reported by Frattaroli [15], although it is within the range of some of the studies reviewed there. The major difference in the current study was the greater length of the intervention. The average study length reported by Frattaroli involved four sessions over 4 days. The current study had 12 sessions spread over 6 months. In addition, in contrast to the studies reviewed by Frattaroli [15], there was no maximum limit set on the length of the expressive writing session that participants undertook. This is a considerable difference and may have contributed to a larger than expected effect in the expressive writing condition. The use of expressive writing over a longer period like this warrants further investigation.

If the expressive writing condition was unexpectedly powerful, then perhaps a comparison with a waitlist control or a weaker intervention would have shown gains for both conditions. In order to test this possibility, we reviewed other studies that used the CES-D to look at change in a control group, in order to see whether the improvement shown over time was a result of a natural tendency for conditions to improve over time. To identify suitable comparison control groups, a systematic review of computer-based psychological treatments for depression was consulted [42]. This identified 19 RCTs and, among these, six studies that used the CES-D, with similar exclusion criteria and recruitment methods to the current study (see Table 6).

**Table 6:** Control group outcomes in studies of computer-based psychological treatments for depression which have used the CES-D.

Study	Follow-up	CES-D at baseline	CES-D at follow-up	Cohen’s d
Baikie et al [ 13]	4 months	30.86 (13.06)	22.02 (14.30)	0.65
Christensen et al [ 43]	6 weeks	21.6 (11.1)	20.6 (11.4)	0.09
Clarke et al [ 44]	16 weeks	31.2 (11.7)	22.7 (12.6)	0.70
Clarke et al [ 44]	32 weeks	--	23 (14)	0.64
Clarke et al [ 45]	16 weeks	28 (13.6)	22.3 (13.1)	0.43
Van Straten et al [ 46]	4 weeks	29.9 (9.2)	26.2 (10.5)	0.37
Warmerdam et al [ 47]	12 weeks	32.1 (9.3)	25.8 (10.4)	0.64

Other than Christensen et al [43], all of the control groups had similar mean initial CES-D scores of approximately 30, as in the present study. At 6-month follow-ups, and mostly over shorter periods, the mean scores in the control groups had dropped to approximately that seen in the current study: 22. The Christensen et al [43] study is slightly different in that participants had lower baseline levels of depression and an attempt was made to control for placebo effects, which was not the case in the other studies.

Thus, the change in CES-D scores seen in the current study in both conditions is likely to reflect a tendency to improve naturally over time, without treatment. The improvements are comparable to the majority of the control groups from other studies cited here. Therefore, while the effect sizes seen in the expressive writing and ISG conditions were medium in size, it is likely that this is the type of effect size that would be seen even in a waitlist control condition. This evidence weakens the notion that either the expressive writing or the ISG condition had any additional effect.

Overall, therefore, there is no evidence that the ISG was effective in ameliorating the symptoms of depression or anxiety. In addition, the ISG had worse attrition and less engagement in comparison to the expressive writing.

### Attrition and Engagement

Attrition rates are frequently high in Web-based studies and, in eHealth interventions, attrition curves are often logarithmic [41]. Still, the attrition rate seen here was especially high in the ISG group. Of the 568 participants randomized to the ISG condition who completed preintervention questionnaires, only 10% (57/568) were classified as engaging with the ISG, and many of these did not use the group much. This may well be a function of the sample, which differed somewhat from those in previous studies. Houston et al [9], for example, who found use of an ISG to be beneficial, recruited existing members of a support group. In the current study, participants were not existing members of an ISG and were asked to take part in forums that were new to them. Because 85% (736/863) of participants had never used an ISG before and 78% (672/863) had never taken part in a face-to-face support group before, they were probably not very aware of what ISGs would be like. This accords with the findings of a naturalistic study, which found similar problems in engaging new users to the same ISG [48], and echoes Eysenbach et al’s [49] warning against ‘recruiting from the street’.

In comparison to the ISG condition, the expressive writing condition had an attrition rate closer to those found in previous Web-based studies [41]. The increase in attrition over the study was also less slow in the expressive writing condition. At 3 months it was 58% (172/295), while at 6 months it was 65% (194/295). The equivalent figures in the ISG were 85% (676/795) and 87% (692/795). It is also worth noting that engagement with the expressive writing tasks was essentially a binary process, participants either engaged or they did not, whereas engagement in the ISG was a more complex process: participants could either contribute by posting material themselves, or they could be actively involved with the group by simply reading other group members’ posts.

The high attrition rates compared with the expressive writing condition was not the only indication that participants were unenthusiastic about the ISG. Across the first 4 to 6 weeks of the study, engagement with the ISG dropped from a mean of once a week to less than once every 2 weeks, remaining at this level or lower for the rest of the study. The same picture came from the data on the amount of time spent accessing the ISG and particularly from the number of posts made. Across all participants in the ISG condition, after the first 2 weeks, even those classified as 'engaged' with the ISG were only posting a mean of approximately one message every 2 weeks.

There are many potential reasons for the low levels of engagement with the ISG, but one that stood out was participants' expectations. Before the study began, and at every measurement point, participants were asked about their expectations of the intervention's usefulness, using the IPQ [35]. Although the other IPQ factors, such as the condition's influence on life, the expected longevity of the condition, and control and understanding of it changed little, expectations of the intervention's usefulness dropped markedly in the ISG group, in comparison with expectations in the expressive writing condition, which remained largely stable over the 3 months. This difference was clear from both baseline to 3 months and between baseline and 6 months. As might be expected it was even clearer when comparing engagers to the ISG with nonengagers. After only 3 months, mean expectations of the intervention's usefulness for nonengagers had dropped to two on the 11-point scale, indicating that they thought it was close to worthless. It is hard to ignore this message that many of the participants in the study expected the ISG to do little for them.

It may have been the case that we underestimated the difficulty of joining an established ISG. It was probably hard for participants to find their way in what to a newcomer is quite an unusual social system. We attempted to mitigate this by briefing participants about the group beforehand and encouraging them to contact the researcher if they were having problems, but this may not have been sufficient to ease their transition into the ISG.

Engagement with the ISG was not predicted by demographic variables, although those reporting higher anxiety were more likely to engage with the ISG and there was a trend in the same direction for depression. However, the expectations in both the engaging and nonengaging groups began at the same level and only dropped after the start of the study. This again suggested that participants did not know what to expect from the ISG and some quickly wrote off the chance of any potential benefits from it. Much the same message came from the satisfaction data. Although engagers were moderately satisfied with the ISG, those who did not engage gave very poor ratings to it.

### Word Count Analysis

The final part of the study examined associations between the type of language used in the ISG and outcome. Of particular interest were positive and negative emotion words and the pronouns used. Only one of the expected correlations was significant, supporting previous findings [50] that the expression of positive emotions was associated with improved psychological health (although this was only found after 3 months, but not after 6 months). Because this part of the research was correlational, it may well be that the use of positive emotion words is a result rather than a cause of lower levels of depression. Nevertheless it may be a useful linguistic marker to assess how participants are reacting in an ISG.

No correlations were found for pronoun use, providing no support for the theory that helping others is beneficial [19] or for the idea that focusing on the self may be detrimental to psychological health in the context of ISGs [51].

### Limitations

The main limitation of the study was the lack of a wait-list control group. We took the view that this would be difficult to implement, as participants could not ethically or practically be prevented from seeking help elsewhere. We instead used previous research on our main outcome variable, the CES-D, to construct a post-hoc quasi-experimental control.

The study involved one particular ISG, PsychCentral, and it is possible that it was not typical of ISGs in general. However, as discussed above, we put considerable effort into its selection, and it appeared to us to be one of the best mental health ISGs available at that time. However, it is remotely possible that PsychCentral might have had some undetected problematic aspects and, in retrospect, it would have been better to offer participants a choice from a shortlist of ISGs that we had approved.

The sample was more highly educated than a typical community sample experiencing depression or anxiety. The greater proportion of women (628/863, 73%) was broadly representative of the higher levels of depression among women.

Engagement was measured using retrospective self-report, which is well known to be subject to potential sources of bias, such as distortions of memory [52]. However, the picture from the self-report data was similar to that from other data sources (ie, the attrition rates and the qualitative comments), all pointing to low levels of engagement with the ISG.

### Conclusions

The findings present a paradox. On the one hand, ISGs for depression and anxiety are thriving, as are those for many physical conditions, which suggests that their many users are benefitting from their experiences. PsychCentral and many other sites maintain high traffic from users who appear to be engaged with and supportive of one another. Some outcome research has found positive results with ISGs for depression [7,10].

On the other hand, the present study, as well as that done by Breuer and Barker [48], found no evidence that ISGs were effective or attractive for potential users who were directed to the groups, in terms of standard outcome variables, and little evidence of benefits in other areas. However, it is important to re-emphasize that our ISG participants were directed by us to the group, rather than seeking it out themselves, and that their engagement was low. So our conclusion that the ISG group showed no evidence of benefit is limited to such ‘recruited from the street’ users [49].

It may well be that people vote with their feet, and that the current satisfied users of anxiety and depression ISGs are a small percentage of the general population. That would mean that interventions by health care professionals or public health campaigns directing individuals to ISGs offer little benefit, unless one can target those who do benefit from ISGs. It may also be the case that even those who engage and presumably appreciate ISGs are not actually benefitting in terms of reduced morbidity.

However, that conclusion does not immediately explain those studies that have found positive results with ISGs for depression, and a larger literature finding positive results for ISGs more broadly. Such heterogeneity in study results may have many explanations. One possibility is that what makes for a successful online intervention and individual experience is subtle and contingent. The effects of technology in health are often not readily determined [53] and an intervention like an ISG depends on a range of complex factors including usability, sociability [54], and the nature of therapeutic relationships.

Until further evidence accumulates, we cannot at present recommend generally directing people with anxiety and depression to internet support as an effective additional intervention. On the other hand, online expressive writing [13] seems to have potential, and its use for people with depression and anxiety warrants further investigation, particularly to examine who engages with it, and who benefits from it, under what conditions.

## Appendix

Multimedia Appendix 1 Demographics for all participants who (1) completed the baseline measures, and (2) were eligible for analysis, by condition.

## Abbreviations

ANOVA: analysis of variance
CES-D: Center for Epidemiologic Studies Depression Scale
GAD-7: General Anxiety Disorder Questionnaire
IPQ: Illness Perception Questionnaire
ISG: Internet support group
LIWC: Linguistic Inquiry and Word Count
LSD: least significant difference
MOSSSS: Medical Outcomes Study Social Support Survey
RCT: randomized controlled trial
SWLS: Satisfaction with Life Scale
