# Supplementary material for: Direction to an Internet Support Group Compared With Online Expressive Writing for People With Depression And Anxiety: A Randomized Trial
Source: JMIR Ment Health. 2016 May 17;3(2):e12. doi: 10.2196/mental.5133 (PMC4887661; doi:10.2196/mental.5133)
Supplement: Multimedia Appendix 1 [file mental_v3i2e12_app1.pdf]

Table 2: Demographics for all participants who (1) completed the baseline measures, and (2) were eligible for analysis, by condition.

		Baseline	Expressive writing completers	ISG completers
Variable		n = 863	n = 101	n = 103
Gender				
	Male	235 (27%)	24 (24%)	26 (25%)
	Female	628 (73%)	77 (77%)	77 (75%)
Age				
	Mean (SD)	34 (12)	37 (12)	35 (12)
	Range	18-75	18-64	18-66
Employment status				
	Full-time	393 (46%)	36 (36%)	47 (45%)
	Part-time	110 (13%)	22 (22%)	16 (16%)
	Student	153 (18%)	9 (9%)	19 (18%)
	Looking after home/family	40 (5%)	4 (4%)	5 (5%)
	Unemployed because of poor health	60 (7%)	13 (13%)	9 (9%)
	Unemployed for other reasons	69 (8%)	11 (11%)	5 (5%)

	Retired	25 (2%)	6 (6%)	1 (1%)
	Other	13 (1%)	0 (0%)	1 (1%)
Education				
	Some high/secondary school	22 (3%)	2 (2%)	1 (1%)
	Completed high/secondary school	86 (10%)	7 (7%)	14 (14%)
	Some college/university	314 (36%)	29 (29%)	24 (23%)
	Degree (eg, BSc BA)	282 (33%)	40 (40%)	37 (36%)
	Advanced degree (eg, Masters, Doctorate)	159 (18%)	23 (23%)	27 (26%)
Ethnicity				
	White	753 (87%)	91 (91%)	94 (91%)
	Asian (including Indian)	38 (4%)	4 (4%)	3 (3%)
	Black	34 (4%)	0 (0%)	1 (1%)
	Hispanic	9 (1%)	1 (1%)	0 (0%)
	Mixed	13 (2%)	4 (4%)	4 (4%)
	Other	16 (2%)	1 (1%)	1 (1%)

Country				
	United Kingdom	578 (67%)	63 (63%)	65 (63%)
	United States	252 (29%)	32 (32%)	32 (31%)
	Canada	33 (4%)	6 (6%)	6 (6%)
Consulted a health care professional				
	Within the last year	393 (45%)	54 (54%)	50 (48%)
	More than a year ago	281 (33%)	35 (35%)	35 (34%)
	Never	181 (21%)	11 (11%)	17 (17%)
	Not sure	8 (1%)	1 (1%)	1 (1%)
Seeing a therapist				
	Yes	191 (22%)	26 (26%)	26 (26%)
	No	659 (76%)	72 (72%)	71 (71%)
	Not sure	13 (2%)	3 (3%)	3 (3%)
Taking medication				
	Yes	257 (30%)	33 (33%)	41 (40%)
	No	601 (69%)	66 (66%)	61 (59%)
	Not sure	5 (1%)	2 (1%)	1 (1%)
Used a face-to-face support group before				
	Yes	180 (21%)	19 (19%)	20 (19%)
	No	672 (78%)	80 (80%)	80 (78%)
	Not sure	11 (1%)	2 (2%)	3 (3%)
Used an ISG before				

	Yes	107 (12%)	16 (16%)	14 (13%)
	No	736 (85%)	82 (82%)	86 (84%)
	Not sure	20 (3%)	3 (3%)	3 (3%)
